# Response of the Public to Preventive Measures of COVID-19 in Iraqi Kurdistan

**DOI:** 10.1017/dmp.2020.233

**Published:** 2020-07-14

**Authors:** Deldar Morad Abdulah, Salem Said Aziz Qazli, Sherzad Khudeida Suleman

**Affiliations:** Community Health Unit, College of Nursing, University of Duhok, Duhok, Iraq; Nursing Department, College of Nursing, University of Duhok, Duhok, Iraq; Witten/Herdecke University, Germany; Community Health and Pediatric Unit, College of Nursing, University of Duhok, Duhok, Iraq

**Keywords:** Coronavirus, environmental exposure, infection control

## Abstract

**Objectives:**

On March 1, 2020, the Kurdistan Region Government (KRG) announced 4 confirmed cases of coronavirus disease (COVID-19). We aimed to explore the response of the public toward the prevention principles against severe acute respiratory syndrome coronavirus 2 (SARS-CoV-2) infection.

**Methods:**

The investigators invited individuals from different geographic areas of Duhok Governorate of Iraqi Kurdistan in March 2020.

**Results:**

The mean age of the participants was 25.74 (16‐95 years). The mean score and prevalence of fear toward SARS-CoV-2 infection was 4.40 of 10 and 81.9%, respectively. A small percentage of participants did not minimize their exposures by reducing close contacts and transmission of respiratory droplets (14.5%) and visited public areas during the epidemic (28.7%). The study revealed that 30.8% of the participants do not use face masks or tissues when they sneeze in public areas. Most of the participants wash their hands when they suspect a possible transmission of the SARS-CoV-2 pathogen (94.6%) and clean or disinfect pathogen contamination-suspected areas at home (84.6%). The study also revealed that some participants (11.2%), due to a lower education, did not visit a medical clinic when they experienced possible symptoms of SARS-CoV-2 infection. Participants agreed with the health policies of KRG against the COVID-19 outbreak (90.8%).

**Conclusions:**

Some individuals do not adhere to preventive measures against SARS-CoV-2 infection.

An epidemic of cases with unexplained low respiratory infection was detected in Wuhan, China. The virus (severe acute respiratory syndrome coronavirus 2 [SARS-CoV-2]) is very contagious and spreads quickly globally. The outbreak of coronavirus disease (COVID-19) was declared by the World Health Organization (WHO) on January 30, 2020. The outbreak has spread to several countries through human-to-human transmission.^1^

Mainly, the virus spreads by respiratory droplets between persons who have close contact with one another (within about 180 cm). The respiratory droplets are produced through coughing and sneezing. A person may be infected by SARS-CoV-2 through touching a surface or object that has the live virus. Accordingly, the virus enters the body through touching the mouth, nose, including eyes. Some patients with COVID-19 have pneumonia in both lungs, multi-organ failure, and mortality in some cases.^2^

The virus has become a serious global health issue, called by the WHO as a “very high” level, on February 28, 2020. Governments across the world are creating countermeasures to alleviate the possible devastating effects of this outbreak. Currently, health organizations coordinate the flows and problem directives and instructions to better mitigate the effects of this global threat.^1^

Currently, there are only supportive therapeutic strategies to deal with this issue. The reduction in infection transmission in the community is the best current strategy as a preventive measure. In China, the progressive decrease in new cases in the last few days is due to aggressive isolation measures.^1^

The Kurdistan Region’s Ministry of Health announced on Friday, February 25, that they are taking all required measures to prevent this new fatal virus within the autonomous Kurdistan Region of Iraq. The Ministry of Health added that it has been following the matters closely in coordination with the WHO to take all necessary measures against this virus. The country was free from known cases of infection at that time. The KRG Ministry of Health (Kurdistan Regional Government) released a statement on Monday, February 28, that there has been no known case of COVID-19 in the region. The KRG put together several new precautionary measures and recommendations aimed to prevent the COVID-19 spread from Iran to the Kurdistan Region.^3^

In March 1, 2020, the Ministry of Health announced 4 confirmed cases of COVID-19 in the Sulaymaniyah Governorate. The infected persons were of a family of 3 persons and a woman who just returned to the Kurdistan Region from Iran. They were quarantined on the border for medical care and supervision. Some confirmed cases of COVID-19 were announced in the capital of the region (Erbil). Accordingly, KRG closed all governmental organizations and the interconnected routes except for medical and security settings. Until now (March 18, 2020), there are no confirmed cases of COVID-19 in Duhok Governorate.^3^

Iraqi and Kurdish officials, including the Ministry of Health, have started to implement precautionary measures for the global, fatal COVID-19 outbreak from griping areas. The precautionary measures made by KRG were the evacuation of Iraqi students from the virus outbreak epicenter in China, an inspection of travelers who visit the region via the Erbil (and Sulaymaniyah) International Airports.^3^

Public health measures, like handwashing, respiratory etiquette, and environmental cleaning at home, are considered to be the crucial public health measures for the protection of individuals and families against respiratory viruses. The abovementioned measures are very effective when COVID-19 is circulating in a community.^4^ Currently, there is no confirmed case of COVID-19 in Duhok Governorate, but there are some persons infected by this virus in the Erbil and Sulaymaniyah Governorates.^3^ Therefore, the public health measures must be implemented to prevent and control the transmission of any respiratory infectious disease, as the infected areas are so close to our community.

The spread of the virus must be controlled; otherwise, we will be facing a fatal global pandemic. Moreover, a world population of 7.8 billion people with different human behaviors, environmental changes, and insufficient global public health measures may quickly turn this obscure animal virus into a real human threat.^5-7^

We explored the response of the public toward prevention measures of control of SARS-CoV-2 infection in Iraqi Kurdistan. Besides, the level of fear of the public toward infection by SARS-CoV-2 was determined in this survey.

## METHODS

### Study Design and Sampling

The persons who live in different geographical locations of Duhok Governorate (Northern Iraq) were invited into this survey. Two of the authors visited the public areas of Duhok Governorate to invite them personally into the study. Some of the participants were included in the study through direct interviews. However, too many people did not visit public areas. At earlier times, the curfew was applied between 6:00 pm and 6:00 am in the region. Besides, the government did not allow people to gather too many people or present as a group in the public. The individuals visited shopping centers for basic needs only. Therefore, we made an online Google form to obtain a sufficient number of participants.

**FIGURE 1:**
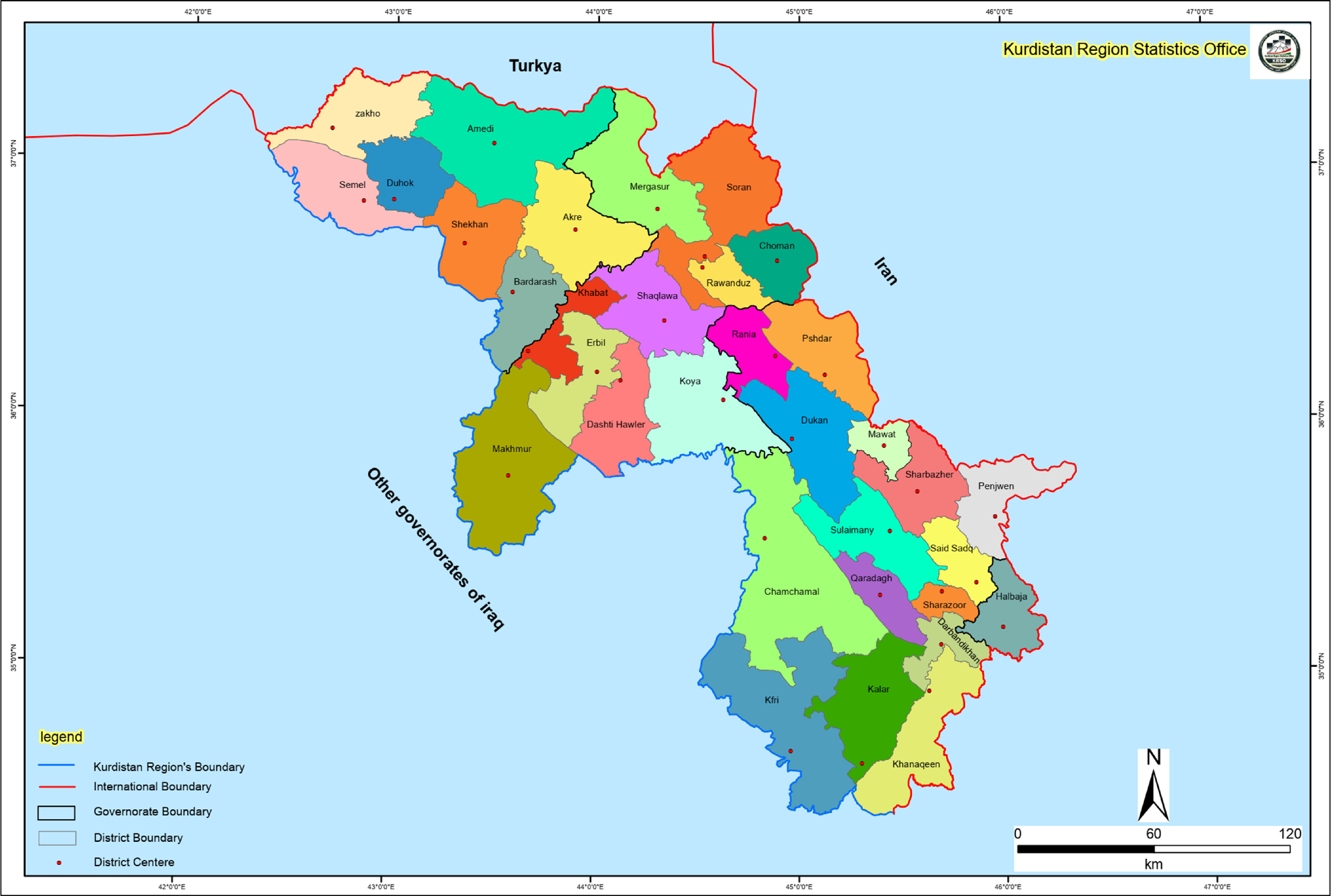
Map of the Districts of Kurdistan Region.^8^

Duhok Governorate consisted of the following districts; Zakho, Amedi, Semel, Duhok, Shekhan, Akre, and Bardarash (Figure 1).^8^ The authors attended the public areas of Duhok and Shekhan Districts to do interviews with people. Duhok is the most populated district inside Duhok Governorate. The investigators could not visit the public areas of other districts due to curfew. We compensated the number of participants by sending an online Google form through social media. However, the sample size of the study may not be the real representative of Duhok Governorate.

The authors invited the individuals on social media by sending an online Google form and personally through a researcher-administered predesigned questionnaire between March 13 and 16, 2020.

### Study Setting

**FIGURE 2:**
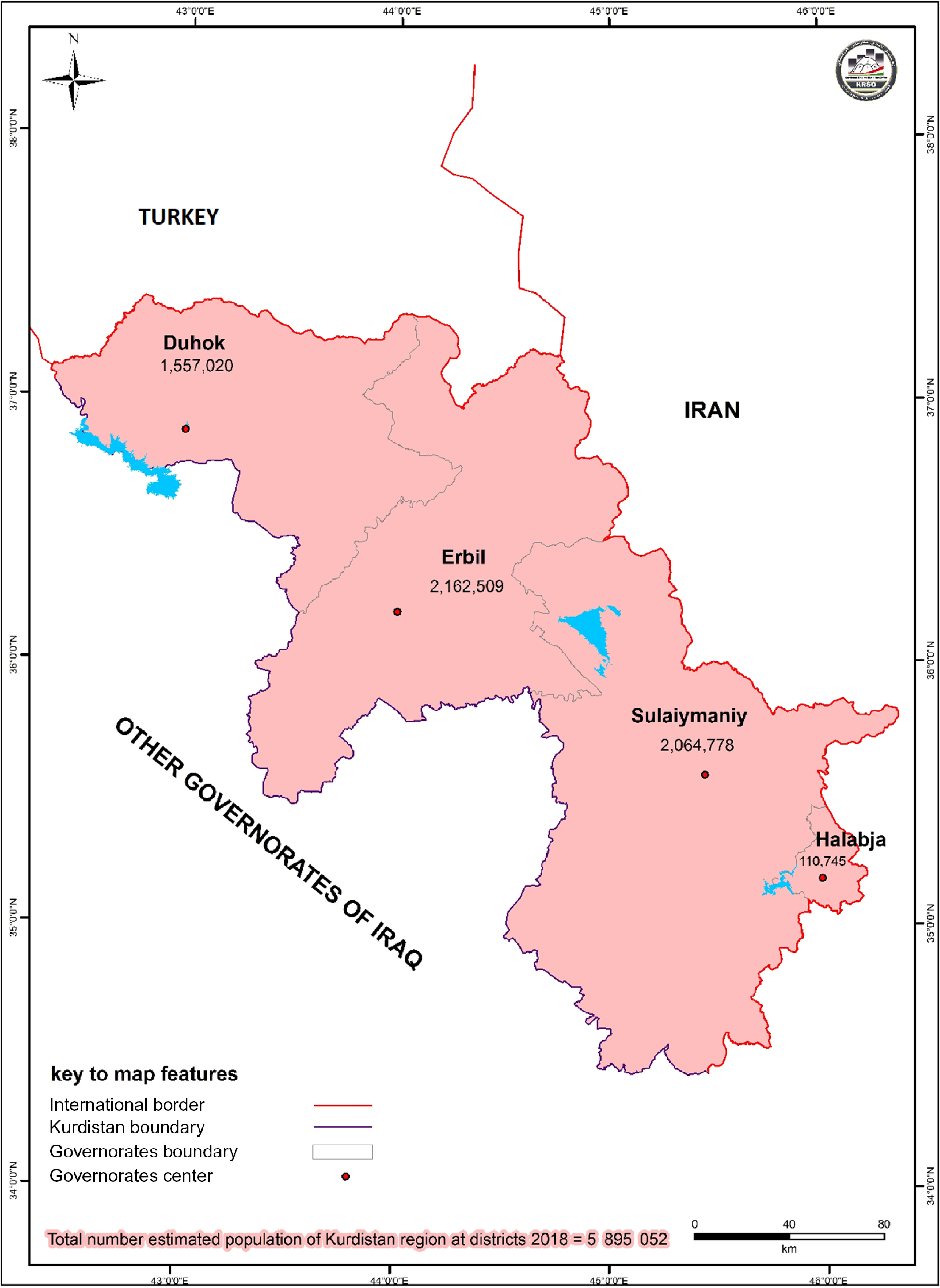
Map of Kurdistan Region With its Estimated Population at Governorate Level – 2018.^8^

Iraqi Kurdistan officially has 4 governorates, including Erbil, Sulaymaniyah, Halabja, and Duhok (Figure 2 ^8^). SARS-CoV-2 entered Iraqi Kurdistan in Sulaymaniyah Governorate on March 1, 2020, in a family and a woman who recently returned from Iran. The confirmed cases were announced in Halabja and Erbil Governorates as well. Duhok Governorate does not share a border with Iran, but it has a border with Turkey, Syria, and other regions of Iraq. Until now (March 17, 2020), the mentioned virus was not recorded throughout the geographic areas of Duhok Governorate. Some cases suspected with the infection of the virus with fever and respiratory droplets from coughing or sneezing were recognized. Still, they were not confirmed to have a positive result of COVID-19.

### Inclusion and Exclusion Criteria

The adult persons of both genders who live in geographic areas of Duhok Governorate, irrespective of sociodemographic aspects, were eligible to participate in this survey. The participants had different education levels, occupations, and religions. The persons who were able to answer the items in the questionnaire were eligible to be included in this study. We invited many individuals from different regions to have a representative sample of the region. Some of the settings were closed during data collection due to the COVID-19 outbreak in the region with few customers. The settings included in the survey were health centers, educational centers, such as universities and schools, and public places, including restaurants, governmental organizations, markets, drivers, and so on. Few persons in shopping centers did not accept our request due to the fear of infection as reported by them. Of the total 1356 forms returned, 8 were blank, 3 had more than 20% of requested information missing, and 2 were from persons under 16 years old. A total of 1343 questionnaires were eligible and included in the study.

### Data Collection and Measures

The questionnaire of the study had 2 parts. In the first part, the authors asked the participants to provide some general information, including age, gender, level of education (categorized as illiterate, high school and under, and college graduate). The participants who completed 2 years (institute degree in this region) and above at a college were categorized as college graduates. Their place of work was categorized as health and non-health settings.

The questions of the study were taken from the prevention and control principles against COVID-19 published by the WHO, titled as “Coronavirus disease (COVID-19) advice for the public.”^9^ We documented whether participants (1) try to minimize their risk of exposure through reducing close exposure to respiratory droplets from coughing or sneezing; (2) visit public areas during the COVID-19 epidemic, like restaurants, markets, and parties; (3) use face masks/tissues when they sneeze in a public location; (4) wash their hands and clothing when they encounter a suspected transmission of the SARS-CoV-2 pathogen; (5) clean or disinfect any pathogen contamination-suspected areas at home; and (6) visit the medical clinic if they experience any symptoms of SARS-CoV-2 infection. Answers were provided in a binary way as either “yes” or “no.”

We added a question to report the agreement of the participants with the current health policies applied by KRG to control the COVID-19 outbreak. Last, participants were asked to rate their fear of being infected with SARS-CoV-2, from zero (“no fear”) to 10 (“extremely fearful”).

### Statistical Analysis

The descriptive characteristics of the participants were presented in mean (SD) or number (percentage). The adherence of participants to the preventive and control measures against the COVID-19 epidemic and their agreement to the Iraqi Kurdistan policies against the spreading of the virus were determined in number and percentage. The outcomes of interest for adherence of the public toward prevention and control of COVID-19 in public were minimizing the risk of exposure, visiting public areas, using face masks/tissues while coughing and sneezing in public, washing hands and clothes, cleaning and disinfecting the suspected areas, and attending the medical clinics in the case of having suspected symptoms of SARS-CoV-2 infection. The level of fear of the participants toward infection was determined in mean (SD). The comparison of a fear level of infection in participants with different characteristics was examined in an independent t-test and a 1-way analysis of variance (ANOVA). The correlation of age with fear of SARS-CoV-2 infection was examined in a bivariate regression analysis. In a regression analysis, the adjustment was made for gender, education, and workplace. The Pearson chi-squared test was performed to examine the association of education level with visiting medical centers in case of having suspected symptoms of COVID-19, minimizing the risk of exposure, and using face masks/tissues during sneezing and coughing in a public location. The significance level of difference was determined in a *P*-value of less than 0.05. The statistical analyses were performed by the Statistical Package for the Social Sciences (SPSS), Version 25 (IBM Corp, Armonk, NY).

### Ethical Considerations

The ethical approval of the present study was obtained from the institutional board of the College of Nursing/University of Duhok on March 9, 2020. The authors asked the individuals to declare whether they agree to participate in the study. The confidentiality of the personal information of the participants was protected in the study.

## RESULTS

The mean age of the participants was 25.74 (SD: 6.45); ages ranged from 16 to 95 years. The participants included males (54.6%) and females (45.4%). The majority of the participants were college-level graduates and above (completing 2 years and above at university, 67.5%) followed by high school graduates and under (18.0%) and illiterate (14.4%). The participants worked in non-medical (70.1%) and medical settings (29.9%), as presented in Table 1.

**TABLE 1:** General Information of Participants

	Age Groups (Years) F(%)									
Characteristics (n = 1343)	16-19	20-29	30-39	40-49	50-59	60-69	70-79	80-89	90-99	Total
**Age** (range: 16-95 years)										
Mean (SD): 25.74 (6.45)	153 (11.4)	760 (56.6)	250 (18.6)	114 (8.5)	38 (2.8)	15 (1.1)	11 (0.8)	1 (0.1)	1 (0.1)	
**Gender**										
Male	57 (37.3)	382 (50.3)	171 (68.4)	76 (66.7)	29 (76.3)	9 (60.0)	8 (72.7)	0 (0.0)	1 (100)	733 (54.6)
Female	96 (62.7)	378 (49.7)	79 (31.6)	38 (33.3)	9 (23.7)	6 (40.0)	3 (27.3)	1 (100)	0 (0.0)	610 (45.4)
**Education**										
Illiterate	22 (14.4)	55 (7.2)	40 (16.0)	39 (34.2)	17 (44.7)	11 (73.3)	9 (81.8)	1 (100.0)	0 (0.0)	194 (14.4)
High school and under	59 (38.6)	96 (12.6)	48 (19.2)	22 (19.3)	15 (39.5)	1 (6.7)	1 (9.1)	0 (0.0)	0 (0.0)	242 (18.0)
College and above	72 (47.1)	609 (80.1)	162 (64.8)	53 (46.5)	6 (15.8)	3 (20.0)	1 (9.1)	0 (0.0)	1 (100)	907 (67.5)
**Working Place**										
Health settings	27 (17.6)	275 (36.2)	66 (26.4)	33 (28.9)	1 (2.6)	0 (0.0)	0 (0.0)	0 (0.0)	0 (0.0)	402 (29.9)
Non-health settings	126 (82.4)	485 (63.8)	184 (73.6)	81 (71.1)	37 (97.4)	15 (100)	11 (100)	1 (100)	1 (100)	941 (70.1)

The study showed that a small percentage of participants did not minimize their exposure by reducing close contacts and transmission of respiratory droplets (coughing, sneezing) (14.5%) and visited public areas during the COVID-19 epidemic (28.7%). The study revealed that 30.8% of the participants do not use face masks or tissues when they sneeze or cough in public areas. However, it revealed that most participants wash their hands and clothing when they suspect a SARS-CoV-2 pathogen transmission (94.6%) and clean or disinfect pathogen contamination-suspected areas at home (84.6%). A small percentage (11.2%) of the participants reported that they do not visit a medical clinic if they feel possible symptoms of COVID-19. Most of the participants (90.8%) did agree with the health policies applied by KRG to control the COVID-19 outbreak in March 2020 (Table 2).

**TABLE 2:** Adherence of Public Toward Prevention Principles to Control COVID-19

	Statistics n (%)	
Characteristics of Participants (n = 1343)	Yes	No
Do you try to minimize the risk of exposure through minimizing close contact with respiratory droplets (coughing or sneezing)?	1148 (85.5)	195 (14.5)
Do you visit public areas during the COVID-19 epidemic, like restaurants, markets, parties, and so on?	385 (28.7)	958 (71.3)
Do you use face masks/tissues when you or someone sneezes or coughs in a public location?	930 (69.2)	413 (30.8)
Do you wash your hands and clothing when there is a risk of SARS-CoV-2 pathogen transmission to you?	1270 (94.6)	73 (5.4)
Do you clean or disinfect the suspected areas of pathogen contamination at home?	1136 (84.6)	207 (15.4)
Do you attend the medical clinic if you experience suspected symptoms of COVID-19?	1193 (88.8)	150 (11.2)
Do you agree with the current health policies applied by Iraqi Kurdistan governed to control the COVID-19 outbreak?	1219 (90.8)	142 (9.2)

The mean score of fear of the participants of getting COVID-19 was 4.40 (SD: 3.34) of 10. The study revealed that 81.9% had a fear of different severities for getting COVID-19. The illiterate individuals had a higher level of fear (mean [M]: 6.53) compared to those with a high school education and under (M: 4.71) and college education and above (M: 3.86; *P* < 0.001). The prevalence of fear of infection in illiterate individuals was 89.2% compared to 83.1% and 80.0% in individuals with a high school education and under, and a college education and above, respectively. However, the level of fear of contracting COVID-19 was not significantly different between both genders and individuals who worked in health and non-health settings: *P* = 0.850 and *P* = 0.865, respectively. The prevalence of fear of infection among individuals who work in health settings and non-health settings was 85.8% and 80.2%, respectively (Table 3).

**TABLE 3:** Comparison of Fear Level of Contracting COVID-19 in Subjects With Different Characteristics

Participants (n = 1343)		Statistics n (%)		
	Mean (SD)	No Fear	Fear with Different Severity	*P*-value
Fear	4.40 (3.34)	243 (18.1)	1100 (81.9)	< 0.001
Gender				0.850*
Male	4.39 (3.20)	119 (16.2)	614 (83.8)	
Female	4.42 (3.50)	124 (20.3)	486 (79.7)	
Education				< 0.001**
Illiterate (Ref)	6.53 (3.49)	21 (10.8)	173 (89.2)	
High school and under	4.71 (3.18)	41 (16.9)	201 (83.1)	
College and above	3.86 (3.15)	181 (20.0)	726 (80.0)	
Working Place				0.865*
Health settings	4.38 (3.09)	57 (14.2)	345 (85.8)	
Non-health settings	4.41 (3.44)	186 (19.8)	755 (80.2)	

*Independent t-test and **1-way ANOVA were performed for statistical analyses.

The correlation of age with fear of contracting COVID-19 showed that the level of fear was increased with increasing age among the public (Table 4).

**TABLE 4:** Correlation of Age With Fear of Infection by COVID-19 in Public

Control Variables		Fear Level of Infection
Age	Correlation	0.098
	Significance (2-tailed)	0.001

The analysis was adjusted for gender, education, and working place.

Bivariate regression was performed for statistical analysis.

The association of educational level with visiting medical centers for suspected symptoms of COVID-19 showed that illiterate individuals (23.2%) substantially were not ready to visit the medical centers compared to those with a high school education and under (6.6%) and a college education and above (9.8%). Similarly, the illiterate individuals were more likely to report that they do not minimize the risk of exposure (35.1%; *P* < 0.001). However, most of the individuals at different educational levels reported that they use face masks/tissues when sneezing or coughing in a public location (*P* = 0.772) (Table 5).

**TABLE 5:** Association of Educational Level With Attending Medical Centers in Case of Having Suspected Symptoms of COVID-19

	Educational Level			
Subjects’ Practice (n = 1343)	Illiterate	High School and Under	College and Above	*P*-Value
Visiting medical centers if experiencing suspected symptoms of COVID-19				
Yes	149 (76.8)	226 (93.4)	818 (90.2)	< 0.001
No	45 (23.2)	16 (6.6)	89 (9.8)	
Minimizing the risk for exposure through minimizing close contact with respiratory droplets (coughing, sneezing, talking)				
Yes	126 (64.9)	189 (78.1)	833 (91.8)	< 0.001
No	68 (35.1)	53 (21.9)	74 (8.2)	
Using face masks/tissues when you or someone sneezes or coughs in a public location				
Yes	136 (70.1)	163 (67.4)	631 (69.6)	0.772
No	58 (29.9)	79 (32.6)	276 (30.4)	

*Note:* Pearson chi-squared test was performed for statistical analysis.

## DISCUSSION

The present survey showed that a small percentage of the participants do not minimize their exposure by reducing close contact with respiratory droplets through coughing and sneezing, and by not visiting public areas during the COVID-19 epidemic. The policies and practices of the Centers for Disease Control and Prevention indicate that the exposure to respiratory pathogens, including SARS-CoV-2, the virus that causes COVID-19, must be minimized. The reason is that large-particle respiratory droplets will spread when someone coughs, sneezes, or talks. Social distancing measures are considered approaches to minimize risk of pathogen exposure to others in a community. The strategies of social distancing include quarantine and self-isolation at the individual level and avoiding crowded areas, school areas, workplace settings, and pubic/mass gatherings at the community level.^10^

Voluntary home quarantine or so-called *self-isolation* has been suggested for an asymptomatic person, because that person has a high risk of transmitting the virus. The health officials should strongly encourage these persons to isolate themselves in the home-setting to avoid contact with healthy individuals for further prevention of virus transmission at the earliest stage of illness.^4^

Voluntary avoidance of crowded places is suggested for an asymptomatic person and a person with a medium risk of exposure to the virus. The strategies are avoiding crowded public spaces and settings in which rapid self-isolation is not feasible upon onset of symptoms. Examples are mass gatherings, such as concerts, sports events, and festivals,^10^ especially the Newroz Festival (a Kurdish festival that starts in the spring, on March 21), and many people may have prepared themselves to visit the tourist places across the region. Newroz is the Kurdish celebration of the New Year holiday. Kurdish Newroz coincides with the Spring Equinox and is a festival celebrating the beginning of spring. It is considered to be the most important festival in Kurdish culture. Typically, the festival is celebrated in the days going to the Spring Equinox, and it is celebrated from March 21 to April 1.

The KRG has requested that people to stay at home. The schools, universities, and governmental organizations, except for medical centers, were officially closed. The persons who return to their homes from trips by planes, trains, and buses, had several contacts with infected persons over their travel.^11^ We suggest that the governments apply the measures that were applied by China to prevent further transmission of this virus. The China measures included isolation, quarantine, social distancing, and community containment.^4,12^

The study revealed that 30.8% of the participants do not use face mask or tissues when coughing or sneezing in public areas. However, most of them wash their hands and clothing when they suspect a possible SARS-CoV-2 pathogen transmission and clean or disinfect pathogen contamination-suspected areas at home. Since SARS-CoV-2 can be spread through contaminated surfaces by respiratory droplets, handwashing with soap and water for at least 20 seconds, or hand sanitizing with alcohol solutions, gels, or tissues to maintain clean hands and fingernails is effective in mitigating the spread of COVID-19. The handwashing must be done before and after preparing and eating food, after using the toilet, after coughing/sneezing into a tissue (or if non-compliant with respiratory etiquette), before and after using a surgical/procedure mask and after removing gloves, after handling body fluid-contaminated waste or laundry, and whenever hands look dirty. Routine cleaning of frequently used surfaces and objects assist the prevention of COVID-19 transmission and mitigate the risk of individuals becoming infected by self-inoculation following touching contaminated surfaces. SARS-CoV-2 has the potential to survive in the environment for several days. Therefore, cleaning frequently touched surfaces will kill the virus, hence prevent transmission of the virus.^4^

The asymptomatic persons should use masks to establish the physical barrier for further prevention of the transmission of the virus because the persons block the dispersion of large-particle respiratory droplets produced by coughing, sneezing, and talking. The face mask is necessary to be combined with some other measures like respiratory etiquette and hand hygiene. Therefore, persons with suspected or confirmed COVID-19 can minimize virus transmission by wearing face masks when they are within close contact with other persons in the home setting.

Some persons traveled to Iran during the COVID-19 epidemic.^13^ The persons who returned by the official borders were quarantined for 2 weeks. However, some other persons returned to the Kurdistan region through the forbidden and unofficial borders with Iran.^14^ These persons went to the communities, including Duhok, and were not quarantined. Members of this community must follow properly the quarantine and self-isolation suggestions to prevent spreading this virus and reduce the number of people with COVID-19. This is the major concern of the authors regarding the COVID-19 spread in this region. The KRG must apply rigid approaches to the persons who are suspected of exposure by infected persons in Iran. The persons who have had contact with COVID-19 patients must be quarantined in special facilities to monitor the onset of symptoms.

Another concern of the authors is that a small percentage of the participants reported that they do not attend the medical clinics if they experience COVID-19 symptoms. We found that most of those persons are illiterate. We have a concern that some of them may consist of the individuals who returned through unofficial borders. The media and health officials need to present the appropriate public health measures for further education of our community. Besides, the persons who work in the medical centers must be informed in advance that the persons may be infectious so they can take the necessary precautions. The lessons that we have to learn from the COVID-19 outbreak in China are that health workers have a 3.8% case fatality rate.^15^

We suggest that the persons who returned to their homes through unofficial international borders be recognized and be subject under mandatory quarantine to help reduce the risk of exposing healthy individuals in the community of infection. However, meanwhile, the careful considerations of the safety of the individual and community, their feasibility of implementations, and the anticipated effectiveness must be taken. In particular, the people in our community have a relatively high level of fear toward infection by this virus.

### Precautionary Measures of Iraqi Kurdistan

Most of the participants did agree with the health policies applied by KRG to control the COVID-19 outbreak in March 2020. The Iraqi Kurdistan implemented some precautionary measures against the global and fatal disease outbreak. The Iraqi Kurdistan used the epicenters to inspect travelers who visit the Kurdistan Region via Erbil and Sulaymaniyah International Airports. Two thermal cameras were established at the airports. The suspected persons were sent to the hospitals for further medical examinations. The intensive care units were designated across the cities in the Kurdistan Region for any possible cases of the virus.^16^

## RECOMMENDATION

We need to be grateful for the aid that we receive from international communities, health, and public health organizations. We suggest that the KRG continue to improve surveillance for this virus across the entire region to mitigate the risk of COVID-19.

## CONCLUSIONS

The present survey showed that most of the members of this community consider public health measures to prevent and control the spread of COVID-19. However, we have a deep concern about those persons who do not willingly use face masks/tissues when they cough or sneeze or do not visit the medical centers when they experience an onset of symptoms for possible COVID-19.

In summary:

- Most of the community members adhered to the preventive measures of the WHO recommendations against SARS-CoV-2 infection.
- Some participants are not ready to visit the medical settings when they experience symptoms of COVID-19.
- Some participants do not use face masks or tissues when they sneeze or cough in public areas.
